# Pathogenic *Leptospira* species in rodents from Corsica (France)

**DOI:** 10.1371/journal.pone.0233776

**Published:** 2020-06-05

**Authors:** Elena Izquierdo-Rodríguez, Ángela Fernández-Álvarez, Natalia Martín-Carrillo, Bernard Marchand, Carlos Feliu, Jordi Miquel, Pilar Foronda, Yann Quilichini

**Affiliations:** 1 Instituto Universitario de Enfermedades Tropicales y Salud Pública de Canarias, Universidad de La Laguna, La Laguna, Canary Islands, Spain; 2 Department Obstetricia y Ginecología, Pediatría, Medicina Preventiva y Salud Pública, Toxicología, Medicina Legal y Forense y Parasitología, Facultad de Farmacia, Universidad de La Laguna, La Laguna, Canary Islands, Spain; 3 UMR SPE 6134, CNRS-Université de Corse, Projet GEM, 20250 Corte, France; 4 Departament de Biologia, Sanitat i Medi Ambient, Facultat de Farmàcia i Ciències de l’Alimentació, Secció de Parasitologia, Universitat de Barcelona, Barcelona, Spain; 5 IRBio, Facultat de Biologia, Universitat de Barcelona, Barcelona, Spain; University of Kentucky College of Medicine, UNITED STATES

## Abstract

Leptospirosis is a worldwide emerging zoonotic disease caused by *Leptospira* species, that in some patients develop severe forms with high mortality. In France, Corsica is the area where the highest incidences have been reported. The present study was focused on the analysis of pathogenic *Leptospira* species in rodents of Corsica, as these micromammals are the main natural reservoirs of the bacteria, in order to identify the circulating species and to locate possible risk focuses of transmission, as no previous study on the presence of *Leptospira* species has been carried out in the island. *Rattus rattus*, *Rattus norvegicus*, *Apodemus sylvaticus* and *Mus musculus domesticus* were captured in the proximity of water sources along Corsica, the detection of pathogenic *Leptospira* species was carried out by amplification of the LipL32 gene. The bacteria were found in all the rodent species analyzed and widely. The general prevalence was 10.4%, reaching the maximum value in Bastia (45%). *Leptospira interrogans* and *Leptospira borgpetersenii* were identified by phylogenetic analysis, but also two sequences which corresponded to an unnamed *Leptospira* species, only previously found in rodents of New Caledonia. The high incidence of human leptospirosis in Corsica could be partially explained by the wide distribution of pathogenic *Leptospira* species identified in this study. Also, the presence of an unknown pathogenic species of *Leptospira* in an area with high prevalence, may be involved in the higher incidence of *Leptospirosis* in this island, however, the zoonotic capacity of this species remains unknown. The results obtained are interesting for public health since all positive samples were found near water sources and one of the routes of transmission of leptospirosis is contact with contaminated water. This information could help the competent entities to take preventive measures, reducing the incidence of human leptospirosis in Corsica.

## Introduction

Leptospirosis is the most prevalent bacterial zoonosis worldwide [1]. It is an emerging infectious disease caused by *Leptospira* spp. spirochetes, which can infect both humans and animals. Pathogenic species cause a nonspecific self-limiting illness in human in most cases, but some patients develop systemic disease and even a severe form, including Weil´s disease and severe pulmonary hemorrhagic syndrome, with high mortality rates, >10% and >74%, respectively [2].

In Europe, the number of human leptospirosis cases has increased probably caused by climate change, the higher sensibility of the diagnostic tests and/or due to the increase in the aquatic activities [3]. Concretely in France, both in continental area and the Mediterranean island of Corsica, human leptospirosis has been reported, in cases which included mild clinical signs, as headache, to severe forms, as meningitis or myocarditis [3]. In 2017, 602 cases of leptospirosis were reported in French metropolitan areas [4].

Although a large variety of mammals have been described as *Leptospira* reservoirs, rodents are believed to be the main reservoirs [1], keeping the bacteria in the kidneys, releasing it through the urine, which constitute a source of infection for humans and other animals [2].

In order to identify the circulating species of *Leptospira* present in Corsica, the aim of the present work was to analyse rodents as the main natural reservoirs of this bacterium, as well as to aware of possible hotspots for the transmission of *Leptospira* species to humans and domestic animals in Corsica.

## Material and methods

**Fig 1:**
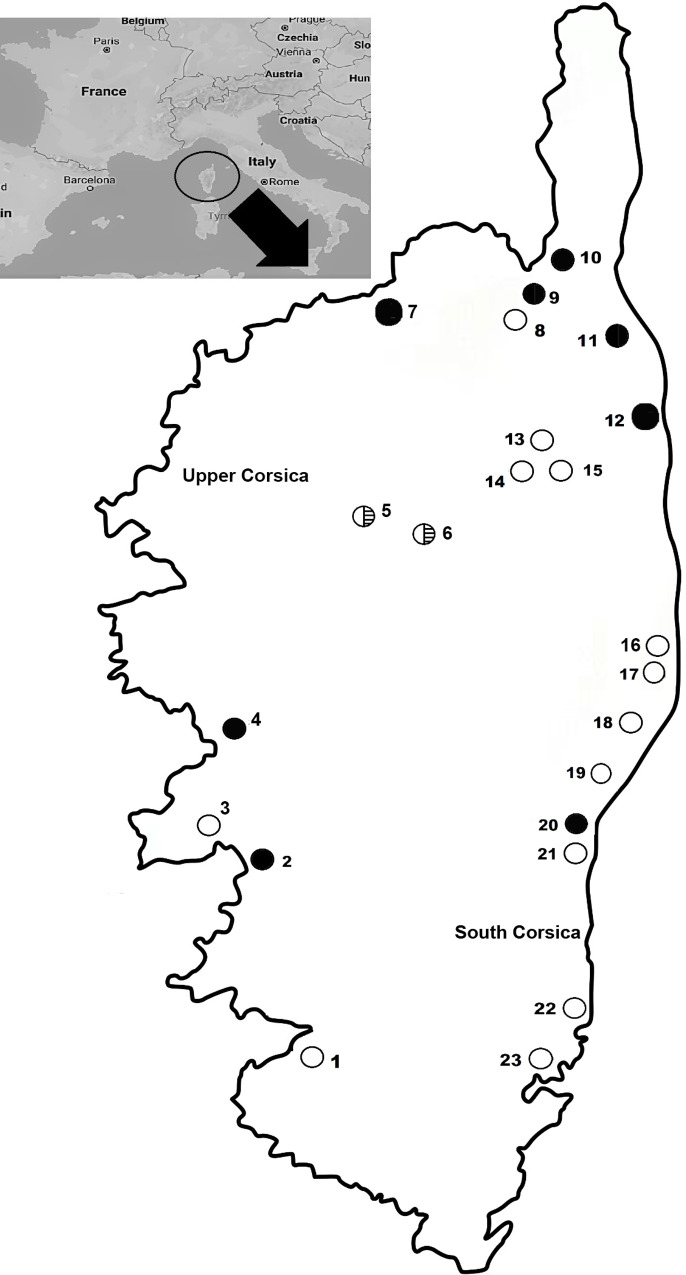
Map of Corsica showing the sampled areas along the island, with positive (●) and negative (○) results for LipL32 presence. (1. Rizzanese, 2. Taravo, 3. Porticcio, 4. Ajaccio, 5. Liamone, 6. Calacuccia, 7. Corte, 8. Foce, 9. Padula, 10. Saint Florent, 11. Tettola, 12. Biguglia, 13. Golo, 14. Chiatra, 15. Matra, 16. Canale di Verde, 17. Terrenzana, 18. Diane, 19. Urbino, 20. Prunelli- di- Fiumorbo, 21. Gradugine, 22. Palo, 23. Pinarellu, 24. Saint Cyprien.). (The original image was took from Wikipedia https://de.wikipedia.org/wiki/Datei:Corsica-locator.svg in which the original author authorized its use for any purpose. From which it was edited by Photoshop CS6).

The study was carried out in 24 locations along Corsica island (France). Between February to June 2016, a total of 115 rodents belonging to the species *Rattus rattus* (80), *Rattus norvegicus* (2), *Mus musculus domesticus* (20) and *Apodemus sylvaticus* (13) were captured alive using Tomahawk and Sherman traps along Corsica (Fig 1). The trap setting was performed at proximity (<500 m) of ponds, river mouths and lakes. Once captured, rodents were euthanized by cervical dislocation or CO_2_ inhalation and bladders containing urine were extracted and stored in absolute ethanol until analyzed. This study was carried out in strict accordance with the recommendations of the guidelines of animal welfare in experimental science and the European Union legislation (Directive 86/609/EEC). The protocol was approved by “Comité de Ética de la Investigación y Bienestar Animal” of Universidad de La Laguna (Protocol Number: CEIBA2018-0330).

Genomic DNA was isolated using a semi-automated extraction system with the QIAamp 96 DNA QIAcube HT Kit (Qiagen Sciences, Inc., Germantown, MD, USA) using the TissueLysser II (Quiagen Sciences, Inc., Germantown, MD, USA), following the manufacturer's instructions. A LipL32 fragment (423 bp), which is present only in pathogenic leptospires, was amplified by PCR according to the method of Levett et al. [5] by using the LipL32-270F (5´-CGCTGAAATGGGAGTTCGTATGATT-3´) and LipL32-692R (5´-CCAACAGATGCAACGAAAGATCCTTT-3´) primers in a MyCycler thermocycler (Bio-Rad, Hercules, CA, USA). *Leptospira interrogans* serovar Icterohemorragiae (RGA strain) was used as a positive control.

The resulting PCR products were sequenced by both sides at Macrogen (Korea), sequences were then aligned by MEGA-X and compared with those available at GenBank by BLAST. New sequences were submitted to GenBank under the accession numbers MN527294-MN527304. A Bayesian analysis of the 354-bp fragment obtained from the LipL32 gene of the species included in this study was set. Sequences from previously published *Leptospira* species were added. The K2 + G was selected as the best-fit model for our sequences and *Leptospira santarosai* (AY461927) was set as an outgroup. The Ngen was set to 30 million and the first 25% trees were discarded.

The confidence intervals (CI) for prevalences were obtained using the Clopper-Pearson exact method (95%). For the comparison of the prevalences obtained in this study Chi square tests were applied setting the P value in 0.05.

## Results

Pathogenic *Leptospira* species were found in 12 out of the 115 rodents analyzed (10.43%; CI: 5.51–17.52), and widely distributed along Corsica (Fig 1). The prevalence was 25% (CI: 66–49.10) for *M*. *m*. *domesticus*, 7.69% (CI: 0.19–36.03) for *A*. *sylvaticus* and 7.32% (CI: 2.73–15.25) for *Rattus* species (5/80 (6.25%; CI: 2.06–13.99) for *R*. *rattus* and 1/2 (50%; CI: 1.26–98.74) for *R*. *norvegicus*) (Table 1). The total prevalence of *Leptospira* sp. in male rodents was 11.48% (CI: 4.74–22.22) (7/61), while in female rodents was 9.43% (CI: 3.13–20.66) (5/53), no significant differences were found when comparing both sexes.

**Table 1:** Distribution of the animals captured in Corsica, divided by rodent species, as well as the location of the positive samples, which were tested by the amplification of the LipL32 gene.

Locations	*Rattus rattus*	*Mus musculus domesticus*	*Apodemus sylvaticus*	Total
	P (%) (+/n)	P (%) (+/n)	P (%) (+/n)	P (%) (+/n)
**Upper Corsica**				
Biguglia	0 (0/3*)	50 (1/2)	-	20 (1/5)
Calacuccia	0 (0/2)	0 (0/1)	0 (0/3)	0 (0/6)
Canale di Verde	0 (0/5)	0 (0/2)	-	0 (0/7)
Chiatra	0 (0/1)	-	-	0 (0/1)
Corte	0 (0/5)	0 (0/3)	0 (0/3)	0 (0/11)
Diane	0 (0/5)	0 (0/1)	-	0 (0/6)
Foce	14.3 (1/7)	0 (0/2)	0 (0/1)	10 (1/10)
Golo	33.3 (1*/3*)	100 (3/3)	-	66.6 (4/6)
Gradugine	0 (0/3)	-	-	0 (0/3)
Matra	0 (0/1)	-	0 (0/1)	0 (0/2)
Padula	0 (0/2)	-	-	0 (0/2)
Palo	33.3 (1/3)	100 (1/1)	-	50 (2/4)
Prunelli-di-Fiumorbo	0 (0/5)	-	-	0 (0/5)
Saint Florent	20 (1/5)	-	0 (0/2)	(1/7)
Terrenzana	0 (0/6)	0 (0/1)	-	0 (0/7)
Tettola	-	-	50 (1/2)	50 (1/2)
Urbino	0 (0/5)	-	-	0 (0/5)
**Total**	**6.5 (4/61)**	**31.2 (5/16)**	**8.3 (1/12)**	**11.2 (10/89)**
**South Corsica**				
Ajaccio	0 (0/4)	-	-	0 (0/4)
Liamone	16.6 (1/6)	0 (0/1)	-	14.3 (1/7)
Pinarellu	-	0 (0/2)	-	0 (0/2)
Porticcio	50 (1/2)	-	-	50 (1/2)
Rizzanese	0 (0/5)	-	-	0 (0/5)
Saint Cyprien	0 (0/1)	-	0 (0/1)	0 (0/2)
Taravo	0 (0/3)	0 (0/1)	-	0 (0/4)
**Total**	**9.5 (2/21)**	**0 (0/4)**	**0 (0/1)**	**7.7 (2/26)**
**TOTAL**	7.3 (6*/82*)	**25 (5/20)**	**7.7 (1/13)**	**10.4 (12/115)**

*: one of the animals was *Rattus norvegicus*; P (%): prevalence of pathogenic *Leptospira* sp.; +/n: number of animals positive for pathogenic *Leptospira* sp./number of animals analyzed.

Geographically, *Leptospira* species were homogeneously distributed along Corsica, and similar prevalence values were found between Upper Corsica (11.24%) and Southern Corsica areas (7.69%), without statistical differences. Nevertheless, the highest prevalence was found in North Corsica, as in Bastia (Biguglia pond and Golo river mouth), Saint Florent (Saint Florent and Tettola) and Foce beach, where the prevalence values were 45.45%, 22.22% and 10%, respectively. In the pond areas of Eastern Corsica (including Terrenzana, Diane, Gradugine, Prunelli-di-Fium'Orbu, Palo and Urbino lakes) the general prevalence was 3.2%, while in Western Corsica the prevalence for Ajaccio (including Ajaccio and Porticcio) and Liamone was 14.3%. However, none of the 30 animals captured in higher inner areas (Corte, Calacuccia, Matra, Chiatra, Canale di Verde and Padula) hosted the bacteria (Fig 1 and Table 1). Significant differences were found when comparing the prevalence of Bastia to Eastern Corsica (Chi square = 5.3619; p = 0.0207).

**Fig 2:**
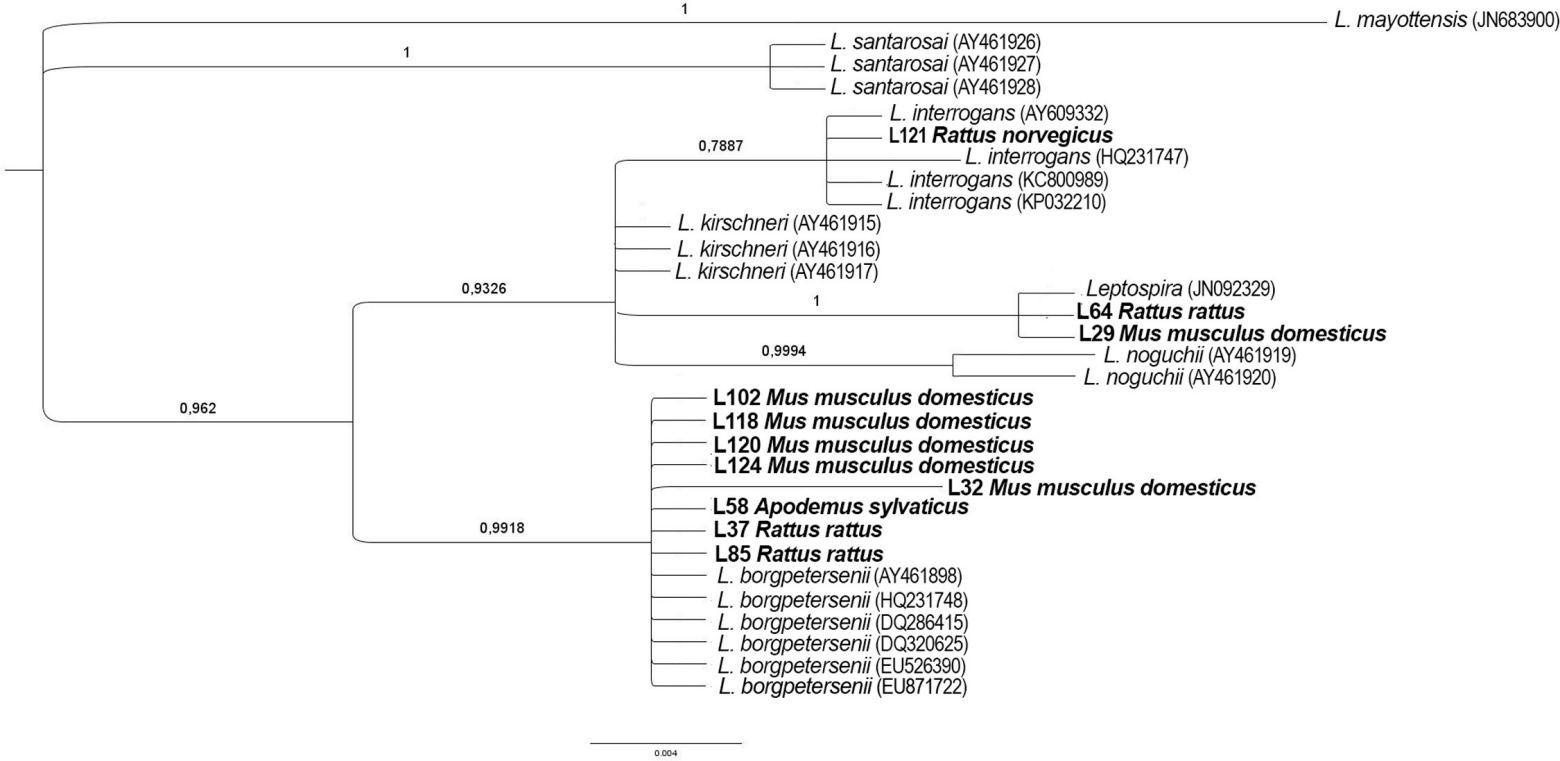
Bayesian analysis based on a 354-bp fragment of the LipL32 gene of *Leptospira* spp. in rodents from Corsica. Sequences belonging to this study are shown in bold and *Leptospira santarosai* (AY461927) was used as outgroup. The K2 + G was selected as the best-fit model. The Ngen was set to 30 million and the first 25% trees were discarded.

Bayesian phylogenetic analysis (Fig 2) showed that eight of the sequences obtained in this study belonging to three host species, *M*. *m*. *domesticus*, *R*. *rattus* and *A*. *sylvaticus*, clustered with *L*. *borgpetersenii*. It is remarkable that two different haplotypes for *L*. *borgpetersenii* were found in Corsica, one of them identical to those obtained in different areas worldwide, such as Brazil (DQ286415) [6], India (EU526390) [7] and the Canary Islands, Spain (HQ231748) [8], among others; while the other (L32 from *M*. *m*. *domesticus* from Palo) showed 5 mutations in the 354bp fragment of the LipL32 gene. Another sequence from *R*. *norvegicus* was identified as *L*. *interrogans*, and the two remaining sequences obtained from *M*. *m*. *domesticus* and *R*. *rattus* were included in a different node as a different species related with *L*. *interrogans* and *L*. *kirschneri*, these two sequences (L29 and L64) showed 97.80% of identity by BLAST analysis with both *L*. *interrogans* and *L*. *kirschneri*, and 100% with an unnamed *Leptospira* species found in New Caledonia (JN092329) [9].

## Discussion

During the last years, the incidence of human leptospirosis has increased along France, reaching the highest value since 1920, 0.9 cases per 100 000 inhabitants. The island of Corsica reported one of the highest incidences of leptospirosis in France on 2017, being 1.87 cases per 100 000 inhabitants, doubling the national mean (0.9) [4].

The presence of *Leptospira* species distributed in wildlife in the proximity of water sources along Corsica may imply a risk of transmission to other animal species and humans, and also may be one of the reasons under the high incidence of human leptospirosis in Corsica, especially considering that one of the many tourist attractions of the island are rivers and beaches. Indeed, in 2017, 8.7 million travellers were registered at Corsican airports [10], so the finding of *Leptospira* species in rodents in near-water locations may imply the transmission of these bacteria not only for locals (farmers, veterinary, fishermen, hunters), but to anyone who visits the island or practice outdoor activities (swimming, kayaking, camping), as outbreaks of human leptospirosis related to recreational activities such as triathlon has been reported in many countries, such as United States [11], Malaysia [12], Germany [13] and Austria [14] in the last decades.

According to the “Institut National de la Statistique et des Etudes Economiques” (INSEE), the total population in Bastia on 2015 was 58098 people, which represents the 17.75% of Corsican population [15]. This agglomeration coincides with the highest *Leptospira* prevalence found in the whole island, where 45.45% of the rodents analyzed were positive for LipL32 gene, setting a possible focus of infection. Besides, this prevalence was found significantly different (p<0.05) when compared to the East of the island, less inhabited [15]. However, only 11 rodents were captured in Bastia area, so further investigation should be done in order to confirm this location as a hotspot for the transmission of *Leptospira* spp.

All positive samples were found in low-altitude locations of Corsica (<400 m), while none of the rodents analyzed in higher locations hosted the bacteria. These results may be correlated with local climate conditions and reveal the possible risk of transmission to human in lower areas presenting high risk of floods. Indeed, despite the complex environmental transmission pathways of *Leptospira*, floods appeared to be among the important drivers on islands [16]. Nevertheless, further investigations should be done on these issues, as the number of samples belonging to this study is limited, and also there are more species that can act as host for *Leptospira* [1]. Also, this study was done in urinary bladders instead of kidneys, which is the organ in which *Leptospira* sp. is the most concentrated, so the incidence for this bacterium may have been under-reported.

Although in humans, significant differences in the prevalence of leptospirosis are observed regarding sex of the individuals [4] no significant differences were found when this comparison was made in rodents. This result may mean that the differences observed in humans are not due to physiological reasons but more related to the fact that men are more likely to have high risk jobs for the transmission of leptospirosis, such as fishermen.

The new *Leptospira* species detected has only been recorded in rodents of New Caledonia [9] and Corsica, islands which are more than 16 thousand kilometres apart. This fact may be explained by the transport of rodents (or other reservoir) between New Caledonia (a French colony) and the European French territory. Further investigations of European rodents should be done in order to determine if this new *Leptospira* species can also be found in rodents of the continent, especially in France. This *Leptospira* species has not been found causing human leptospirosis, so its zoonotic capacity is unknown, nevertheless, it expresses the LipL32 protein, which is involved in pathogenesis of the genus *Leptospira* [17]. Also, taking into consideration that Corsica has reported a higher prevalence of human leptospirosis than the national mean [4], the genotypes of human leptospirosis occurring in Corsica should be studied in order to determine if this *Leptospira* species may be somehow responsible of the high incidence of human leptospirosis in the island.

It is remarkable that both *Leptospira* species found in Palo pond are different to those normally identified in rodents, as one of them was the new *Leptospira* species from New Caledonia and the other correspond to a *L*. *borgpetersenii* haplotype never recorded before. This situation may be due to the isolation that this area experienced during the barbarian invasions, when the villages close to the sea were abandoned and the Corsican population hid in the mountains. It is recorded that during those times other diseases, such as malaria and plague, were present in the island [18]. The presence of rats probably infected with *Leptospira* species and the isolation could be the reason under the finding of the new haplotype, and the constant attacks that the island suffered may have brought rodents from different areas of the world. The presence of new haplotypes of *Leptospira* species due to isolation is also present in the Canary Islands [8], an Atlantic archipelago where the *L*. *interrogans* haplotype found in rodents (HQ231747) (Fig 2) also showed differences when compared to all the sequences uploaded to the GenBank data base.

Considering that early diagnostic of leptospirosis for establishing an effective treatment is vital for patients, the finding of possible risk focuses could help clinicians to familiarize on early recognition of the disease. Also, preventive measures should be applied in order to reduce the high incidence of human leptospirosis in Corsica, such as rodent control and vaccination, which is advised by the French calendar of vaccination and vaccine recommendation for workers in closed and repetitive contact with contaminated environments, such as fishermen, or people practicing ludic activities as rafting, diving, triathlon and other sports which imply humid environments [19].
